# Supplementary material for: Nesfatin-1 in Human Milk and Its Association with Infant Anthropometry
Source: Nutrients. 2022 Dec 30;15(1):176. doi: 10.3390/nu15010176 (PMC9824050; doi:10.3390/nu15010176)

Figure S1. Directed acyclic graph showing hypothesized interactions with infant sex, birth weight, and milk formula supplementation

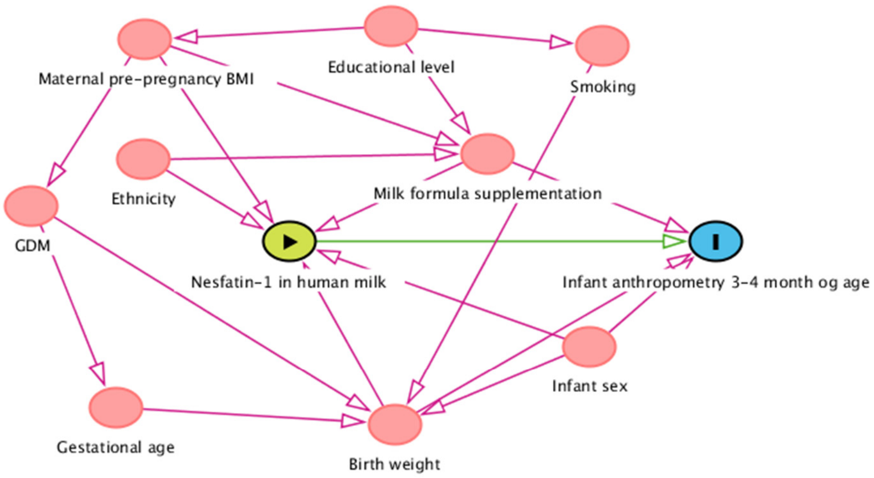

Supplement: Supplementary file 1 [file nutrients-15-00176-s001.zip › Figure S1.pdf]
