# Supplementary material for: Nesfatin-1 in Human Milk and Its Association with Infant Anthropometry
Source: Nutrients. 2022 Dec 30;15(1):176. doi: 10.3390/nu15010176 (PMC9824050; doi:10.3390/nu15010176)
Supplement: Supplementary file 1 [file nutrients-15-00176-s001.zip › Table S1.pdf]

Table S1. Unadjusted multivariate linear regressions for log[nesfatin-1] and anthropometrical values

**Explanatory Variables**	**Log[nesfatin] ng/mL**
**Outcome meassure**	β (95% CI) **P**
**Δ Weight since birth (g)**	24.16 (-313.7-362.0) **0.89**
**Δ WAZ since birth (SD)**	0.19 (-0.20-0.59) **0.34**
**Δ Weight since birth per day (g)**	0.68 (-1.81-3.18) **0.59**
**Lenght at sampling (cm)**	-0.30 (-1.27-0.67) **0.55**
**Weight at sampling (g)**	-88.0 (-489.1-313.08) **0.66**
**Abdominal circumference (cm)**	-0.05 (-1.18-1.08) **0.93**
**Triceps skinfold thickness (mm)**	-0.02 (-0.65-0.61) **0.95**
**Subscapular skinfold thickness (mm)**	-0.11 (-0.57-0.36) **0.65**
**WAZ (SD)**	-0.07 (-0.51-0.36) **0.74**
**HAZ (SD)**	-0.09 (-0.48-0.29) **0.63**
**WHZ (SD)**	-0.007 (-0.39-0.37) **0.97**
**BMIZ (SD)**	-0.021 (-0.42-0.38) **0.92**

The critical value of p at a false discovery rate of 5% was 0.004.
